# Does the Health Kuznets Curve hypothesis hold for Saudi Arabia? A quantile ARDL analysis of disability-adjusted life years and their burden components

**DOI:** 10.3389/fpubh.2025.1667798

**Published:** 2025-12-05

**Authors:** Ousama Ben-Salha, Mehdi Abid, Nasareldeen Hamed Ahmed Alnor

**Affiliations:** 1 Humanities and Social Research Center, Northern Border University, Arar, Saudi Arabia; 2 Department of Finance and Investment, College of Business, Jouf University, Skaka, Saudi Arabia; 3 Department of Accounting, College of Business, Jouf University, Skaka, Saudi Arabia; 4 King Salman Center for Disability Research, Riyadh, Saudi Arabia

**Keywords:** Health Kuznets Curve, disability-adjusted life years, years of life lost, years lived with disability, economic growth, Saudi Arabia, quantile ARDL

## Abstract

Even though the drivers of public health have been extensively investigated, there is a lack of evidence on what contributes to the public health burden in developing countries. This research bridges this gap by testing the Health Kuznets Curve hypothesis in Saudi Arabia, while accounting for the conditional effects of economic, environmental, and social factors within the Grossman health production framework. Three measures of public health burden are considered: Disability-Adjusted Life Years (DALYs) as an overall indicator, Years of Life Lost (YLLs) as a proxy for premature deaths, and Years Lived with Disability (YLDs) as a proxy for morbidity. Using annual data covering the period 1990–2021, this study employs the Quantile Autoregressive Distributed Lag model to investigate the nonlinear effects of key determinants across different quantiles of public health burden. The results reveal an inverted U-shaped association between income and public health, supporting the Health Kuznets Curve hypothesis for all quantiles of DALYs, with a turning point ranging between $20,543.805 and $21,459.450. The findings also reveal an inverted U-shaped relationship for YLLs and YLDs. However, the turning points for YLLs are slightly higher than those associated with YLDs, suggesting that economic growth reduces non-fatal health burdens before it reduces premature deaths. Finally, the findings reveal that GHG emissions and unemployment deteriorate health outcomes across all quantiles, while the impact of globalization is mixed.

## 1 Introduction

An increased debate among policymakers and international organizations centers on the role of economic growth in public health outcomes. Economic growth is considered a key driver of public health and is essential for promoting good health and well-being, as outlined in Sustainable Development Goal 3 (2–4). A common consensus among scholars holds that the better public health outcomes seen in developed countries compared to their developing counterparts are mainly due to differences in income level (5, 6). This disparity became particularly evident during the COVID-19 pandemic. Countries with higher-income levels, often a result of sustained economic growth, responded more effectively to the pandemic, benefiting from greater access to healthcare infrastructure and vaccine distribution. In contrast, the health crisis has had disproportionately severe repercussions on low-income countries, thereby highlighting the systemic fragility of their health infrastructures, partly due to their constrained economic capacity. This disparity raises an important inquiry: To what extent does economic growth influence public health outcomes?

Economic growth can either improve or deteriorate public health outcomes. On the one hand, increased income is associated with improved health by promoting better living conditions and facilitating access to quality healthcare and medical services (2, 7). On the other hand, higher income may adversely affect health by fostering lifestyle-related health risks, including obesity, stress-induced conditions, and non-communicable diseases (8). The detrimental health repercussions of modern economies also include increased consumption of processed foods, mental disorders, and environmental degradation from industrialization and increased economic activity. The previous discussion suggests that the previous literature reached conflicting findings regarding the influence of economic growth on health. The previous discussion also supports a linear connection between growth and public health, implying that economic growth either enhances or deteriorates it. Nonetheless, recent research, including Windarti et al. (9) and Nagano et al. (10), underscores that the relationship between public health and economic growth may follow a nonlinear pattern. The nonlinear association is embodied by the so-called Health Kuznets Curve (HKC) hypothesis. The HKC, which draws an analogy to the Environmental Kuznets Curve describing the income-environment relationship, postulates that health initially deteriorate as income increases, primarily due to factors such as urbanization, adverse lifestyle shifts, and increased environmental degradation. Health outcomes are then expected to improve once income exceeds a given threshold, enabling investments in healthcare systems and environmental protection. Despite its relevance and potential significance, HKC has received relatively little attention, as most previous studies relied on linear assumptions. The limited empirical literature on the nonlinear relationship between economic growth and public health suggests a pressing need for further research to be conducted.

This research adds to the existing body of knowledge by exploring the nonlinear association between economic growth and public health in Saudi Arabia between 1990 and 2021. This topic holds particular importance in Saudi Arabia for at least two key reasons. First, Saudi Arabia has made substantial improvements in health indicators in recent years. According to the World Health Organization (11), life expectancy at birth in Saudi Arabia increased from 70.5 years in 2000 to 76.5 years in 2021. Healthcare coverage also expanded from 86% in 2019 to 96.4% in 2023, while the Healthcare Services Quality Index reached 59% in 2023, exceeding the target level of 49% (12). Concurrently, Saudi Arabia has achieved relatively good economic performance over the last decades, mainly driven by its natural resource endowments, strategic investments, and structural reforms (13). In 2022, Saudi Arabia achieved the highest growth rate among G20 nations (14) and was ranked 19th out of 195 economies in terms of GDP in 2023 (15). In light of the foregoing discussion, analyzing the growth-health nexus in Saudi Arabia holds a paramount significance.

This contribution of this study to the literature on the economic growth-health relationship lies in several key areas. First, it builds on previous empirical frameworks by employing the Disability-Adjusted Life Years (DALYs) as a proxy of public health burden. Indeed, previous research explored the linkage between economic growth and health through various lenses. For example, Grecu and Rotthoff (16) and Windarti et al. (10) examined how economic growth influences obesity rates, while Nagano et al. (11) focused on cardiovascular health. Meanwhile, Fotourehchi and Çalışkan (17) and Zhang et al. (18) utilized life expectancy at birth. Since conventional health indicators typically capture limited aspects of public health, this study employs DALYs to provide a better health metric that reflects the overall health burden. Indeed, DALYs represent the total number of years lost due to ill-health, disability, or premature death (19). According to the World Health Organization (20), one DALY corresponds to one lost year of healthy life. Second, this study adds to the literature by considering not only DALYs as a metric of public health, but also their two core components, Years of Life Lost (YLLs) and Years Lived with Disability (YLDs). While DALYs represent the total health burden, they are computed as the sum of the years of life lost due to premature mortality (YLLs) and the number of years of healthy life lost due to disability (YLDs). The two components capture different aspects of health loss. YLLs measure the burden from premature death by estimating the years lost compared to a standard life expectancy, while YLDs represent the burden of non-fatal health issues by quantifying the time spent with disease or disability. Considering these components allows us to check how economic growth affects different types of public health outcomes. It also allows assessing whether the HKC hypothesis holds for mortality-related and morbidity-related health burdens. Third, the study contributes methodologically to the literature by exploring the HKC hypothesis using the Quantile Autoregressive Distributed Lag (QARDL) model proposed by Cho et al. (21). Unlike conventional OLS-based estimation techniques that focus on mean effects, the QARDL model captures the effects across different conditional distributions of public health. Such an analysis allows for identifying whether economic growth affects health outcomes at various levels of public health (low, medium, and high). By considering distributional heterogeneity, the study can reveal whether the growth–health relationship varies across different levels of public health. This is particularly important for policymakers, as it reveals the effects of economic growth on health outcomes in various public health situations and whether its benefits are concentrated under specific health conditions. Finally, to the best of the authors’ knowledge, no prior investigations examined the effects of economic growth on public health in Saudi Arabia within the framework of the HKC hypothesis. This research addresses the limited evidence on the HKC in developing countries by focusing on Saudi Arabia, a rapidly growing, resource-rich economy where the HKC hypothesis remains untested. From a policy standpoint, the results of this research may provide valuable insights for Saudi policymakers seeking to balance economic expansion with improvements in public health.

The structure of this study is as follows. In Section 2, we provide an overview of the previous literature, while Section 3 outlines the dataset and econometric settings. Section 4 presents the empirical outcomes of the ARDL and QARDL models. The findings are then discussed in Section 5. Finally, Section 6 concludes.

## 2 Related literature

### 2.1 Economic growth and public health: theoretical insights

Many theoretical frameworks have explored the pathways via which growth may affect health. Some of them analyzed the linear influence of economic growth on health, while others have focused on its nonlinear effects. Theories assuming linear effects suggest either positive or negative impacts, i.e., economic growth either improves or worsens public health. Morgado (22) explains the growth-health linkage through the absolute income hypothesis, which postulates that low income leads to poor health outcomes, while higher income results in better health outcomes. The absolute income hypothesis suggests a linear impact of income on public health. The Modernization Theory, initially developed by Rostow (23), highlights that, at certain stages of development, growth may lead to social and institutional modernization. Among others, this could improve healthcare services, support public health, and promote the overall well-being. The theories of public finance and welfare state (24, 25), on the other hand, highlight the role of economic growth in providing financial resources to develop public health infrastructure. This could lead to improved healthcare services and better health outcomes.

Economic growth may also be detrimental to public health, possibly due to potential lifestyle and societal changes resulting from improved income due to economic growth. This phenomenon can be explained through the concept of Diseases of Affluence, which indicates that cardiovascular diseases and other non-communicable disease risks increase as societies develop economically. Indeed, an increase in income often leads to higher consumption of processed foods, mental health issues, and environmental harm caused by industrialization and increased economic activity (26–29). The Diseases of Affluence is linked to the Epidemiologic Transition Theory developed by Omran (30). It highlights that improvements in economic growth may lead to a shift from infectious to chronic diseases due to changes in habits. Despite the above theories and frameworks discussing the growth-health nexus, they have been criticized for their deterministic assumptions, as they overlook critical issues, such as inequality, governance, and the distributional aspects of growth.

Alternative theoretical frameworks have instead argued that the effects of economic growth on health is nonlinear and depends upon the development stage. The Environmental Kuznets Curve (EKC) developed by Grossman and Krueger (31) is the basis for this assumption. It indicates that environmental degradation and the associated health risks initially increase with growth but then decline once this latter exceeds a given threshold. This gives an inverted U-shaped relationship. Building on this hypothesis, the Health Kuznets Curve extended the conceptual framework to public health, suggesting a U-shaped linkage between growth and health. Although not formally proposed as a separate theory, empirical research conducted by many scholars, including Grecu and Rotthoff (16) and Costa-Font et al. (32), supports the HKC hypothesis. The HKC hypothesis suggests a nonlinear association between economic growth and public health. During the early stages of economic development, public health tends to deteriorate, but improves once a given income is exceeded. The first stage of economic growth is characterized by industrial expansion, environmental degradation, and underdeveloped health infrastructure. This may contribute to increased disease burden at this stage. As economies grow and GDP reach a given income level, more investments in healthcare infrastructure, improvements in education, and environmental awareness lead to a positive impact on public health.

### 2.2 Empirical evidence

#### 2.2.1 Linear effects of economic growth on public health

The prior empirical literature investigated the linear and nonlinear impacts of economic growth on health outcomes. Regarding the linear effects, most research supported the view that economic growth positively affects public health, while relatively few studies have identified potential adverse effects. A recent systematic literature review by Yeboah et al. (33) examining 1,167 studies published between 2000 and 2022 and included 18 that met the eligibility criteria revealed that over 60% of studies reported a positive impact of economic growth on health. Morgado (22) analyzed the impact of economic growth on public health in Portugal between 1960 and 2005 using the VAR and Granger causality tests. GDP is found to increase life expectancy, while the reverse causation has not been supported. Using the quantile mixed model, Bai et al. (34) reported a positive effect of GDP on life expectancy in 65 Belt and Road countries over the period 2000–2014. The reported results are valid for both women and men in all countries. However, the effects are more substantial in countries with better initial life expectancy. Cardona et al. (35) explored the influence of economic recession on under-five mortality across 129 countries. The multilevel mixed effects regression revealed that downturns in low- and middle-income countries increase child mortality due to disrupting nutrition and lack of access to healthcare services. Majeed and Ozturk (36) also analyzed the linkage between health and economic, social, and environmental indicators. The analysis indicates that increased growth is positively associated with improved population health. Likewise, Akter et al. (37) examined the determinants of under-five mortality in G7 using monthly data between 1971 and 2021. Applying Grossman’s (1) health production function and Driscoll-Kraay robust standard error technique, the study found that economic growth significantly reduces under-five mortality. Kanat et al. (7) analyzed the short- and long-run repercussion of economic growth on life expectancy in Kazakhstan using the linear ARDL model. The results shows that economic growth has a positive short- and long-run impact on life expectancy. Recently, Rajapakse and Jayathilaka (38) analyzed the effects of growth on under-five mortality rates in Sri Lanka using the multiple linear regression model, while Zhang et al. (18) examined the influence of economic growth on public health in Ghana using the Quantile-on-Quantile regression. The outcomes indicate that growth positively affects life expectancy, with the impact being more pronounced at higher quantiles. This indicates that the health benefits of growth intensify in the presence of higher levels of income and life expectancy.

Studies revealing adverse effects of economic growth on public health are relatively scarce. For example, Adeosun et al. (9) employed the ARDL model to examine the effects of economic growth, inflation, and population size on mortality rates in Nigeria over the period 1991 to 2019. The findings indicate a positive short-run relationship between GDP and mortality, indicating that economic expansion is associated with a deterioration in public health. Different results are reached by Duque et al. (39) and Hill et al. (40), who concluded no significant effects of income on life expectancy in Brazil and the US, respectively. Wang and Ren (41) also reported an insignificant association between GDP and life expectancy among the Chinese population. Despite growing interest in the topic, only two recent studies examined the linear relationship between economic growth and public health in Saudi Arabia. Omri et al. (42) applied fully modified OLS to check the impact of GDP on health in Saudi Arabia for the 1990–2020 period. The findings indicate a positive connection between GDP and both infant mortality and DALYs. Similarly, Islam et al. (43) explored the association between health outcomes and GDP in Saudi Arabia, reporting that infant mortality, road traffic mortality, and healthcare expenditure decline as GDP grows.

#### 2.2.2 Nonlinear effects of economic growth on public health

The nonlinear effects of economic growth on public health have been generally investigated in the lens of the HKC hypothesis. The HKC hypothesis suggests an inverted U-shaped association between economic growth and public health. Different functional forms of the HKC hypothesis have been investigated in the literature, using various dependent variables to capture health outcomes. The Obesity Kuznets Curve hypothesis, introduced by Grecu and Rotthoff (16), establishes an inverted U-shaped relationship between income and obesity, indicating that obesity rates rise with income at lower levels of development but decline once a higher income is reached. Windarti et al. (10) generalized the Obesity Kuznets Curve hypothesis and investigated its validity for a sample of 130 nations between 1975 and 2010. Other related hypotheses, such as the Heart Kuznets Curve proposed by Nagano et al. (11), have also been investigated in the literature. This framework extends the Obesity Kuznets Curve by examining the nonlinear connection between growth and cardiovascular health. Limited studies investigated the HKC hypothesis. For example, Fotourehchi and Çalışkan (17) conducted an empirical analysis of 60 developing countries from 1995 to 2010, using life expectancy at birth and infant mortality to measure health outcomes. The fixed effects model did not reveal a consistent HKC pattern. Costa-Font et al. (32) also examined the HKC using data from the European Community Household Panel survey. Unlike Fotourehchi and Çalışkan (17), the study confirmed the validity of the HKC, with a turning point ranging between $26,000 and $38,700. Recently, Niu et al. (6) analyzed the threshold effects of economic growth on health expenditure, as an indicator of public health, in 30 provinces between 2000 and 2017. The authors confirmed the presence of a threshold effect, where economic growth has a positive and significant impact on health outcomes before and after the threshold level. However, economic growth exerts a higher positive effect on public health before the threshold level (1.167) than after the threshold level (1.146). This confirms the presence of nonlinear effects. Despite providing evidence of nonlinearity, the main drawback of the study is that is only considers heath expenditure as a public health metric, which may not reflect the real public health situation. Finally, Xue et al. (44) explored the nonlinear impacts of economic growth on mortality due to air pollution in China between 2002 and 2021. The authors revealed a U-shaped relationship, as economic growth initially reduces mortality, and then increases it, with an estimated turning point of 99,708 CNY per person.

### 2.3 Research gaps and contributions

The previous review suggests many gaps in the existing studies. First, most previous studies focused on the linear connection between economic growth and public health, yielding mixed results. This limitation highlights the importance of exploring potential nonlinear dynamics, where the impact may vary depending on the stage of development. Additionally, previous studies relied on specific health indicators, particularly life expectancy and mortality rate, which capture particular dimensions of public health. Finally, OLS-based techniques have been generally employed to estimate the effects of economic growth on health. Some exceptions include Bai et al. (34) and Zhang et al. (18), who used quantile mixed model and quantile-on-quantile regression, respectively. To address these gaps, this study investigates the nonlinear effects of economic growth on public health in Saudi Arabia through the lens of the HKC hypothesis using DALYs as a health metric. In addition, the study employs not only DALYs but also their two components (YLLs and YLDs) as dependent variables. While they represent the overall health burden, they differ in the aspects of health they capture: YLLs reflect premature mortality, whereas YLDs account for the non-fatal health outcomes by measuring the years lived with illness or disability. This distinction enables a more comprehensive understanding of the impact of economic growth on public health outcomes. Finally, the study performs the QARDL model, which enables assessing the impact of growth on public health across different levels of public health (low, moderate, high).

## 3 Materials and methods

### 3.1 Model specification

This research examines the impacts of income and public health based on the Health Output (HO) model developed by Grossman (1). Grossman’s model conceptualizes health as a form of durable capital stock that produces healthy time and depreciates over age. Individuals are modeled as both producers and consumers of health, who invest in it through medical care, nutrition, and lifestyle choices to maximize their lifetime utility. The health production function describes the relationship between health inputs and an individual’s health outcomes (37). The general form of the underlying health production function is presented in Equation (1):


HO=F(HI) (1)

where HO represents the health output and HI denotes health inputs.

Following Majeed and Ozturk (36) and Omri et al. (42), this study categorizes health inputs into three main components: economic (ECON), environmental (ENV), and social (SOC) factors. Accordingly, the health production function can be specified as shown in Equation (2):


HO=∫(ECON,ENV,SOC) (2)

In Equation 2, the dependent variable—Health Output (HO)—is measured using three comprehensive public health indicators: Disability-Adjusted Life Years (DALYs), which reflect the overall burden of disease, Years of Life Lost (YLLs), which capture premature mortality, and Years Lived with Disability (YLDs), which represent non-fatal health outcomes or morbidity.

The Health Output model has gained popularity in recent years, making it appropriate for the analysis of the health impact of socioeconomic factors, including economic growth (45). For the economic dimension, we include gross domestic product (GDP) and its squared term (GDPSQ) to examine both the direct and indirect (nonlinear) effects of economic growth on health outcomes, allowing us to test the validity of the HKC hypothesis. The model incorporates greenhouse gas (GHG) emissions as a key indicator of environmental degradation. To account for the social dimension, the unemployment rate (UNEM) is included as a proxy for labor market conditions and broader aspects of social well-being. Beyond the three aforementioned variables, the specification also incorporates economic globalization (KOF) as an additional economic variable that may affect public health. The inclusion of the economic globalization index allows to capture whether the degree of integration into the global economy has had influenced public health in Saudi Arabia. The health impacts of globalization have attracted growing attention in recent years (46–48, 69). However, findings are mixed. Some studies concluded that globalization improves public health via improved access to medical technology, knowledge transfer and food availability. On the other hand, it may deteriorate public health through increased spread of infectious diseases and the exchange of unhealthy products (49, 50).

To test the validity of the HKC hypothesis for the three dependent variables mentioned above, the extended health outcome function can be expressed as shown in Equations (3–5):

Model 1: Overall disease burden


$$\begin{array}{l} \mathrm{DAL}Y_{t}=\alpha_{0}+\alpha_{1}\mathrm{GD}P_{t}+\alpha_{2}\text{GDPS}Q_{t}+\alpha_{3}\mathrm{GH}G_{t}\\ +\alpha_{4}\mathrm{UNE}M_{t}+\alpha_{5}\mathrm{KO}F_{t}+\varphi_{t} \end{array}$$ (3)

Model 2: Mortality


$$\begin{array}{l} \mathrm{YL}L_{t}=\beta_{0}+\beta_{1}\mathrm{GD}P_{t}+\beta_{2}\text{GDPS}Q_{t}+\beta_{3}\mathrm{GH}G_{t}\\ +\beta_{4}\mathrm{UNE}M_{t}+\beta_{5}\mathrm{KO}F_{t}+\varepsilon_{t} \end{array}$$ (4)

Model 3: Morbidity


$$\begin{array}{l} \mathrm{YL}D_{t}=\lambda_{0}+\lambda_{1}\mathrm{GD}P_{t}+\lambda_{2}\text{GDPS}Q_{t}+\lambda_{3}\mathrm{GH}G_{t}\\ +\lambda_{4}\mathrm{UNE}M_{t}+\lambda_{5}\mathrm{KO}F_{t}+\omega_{t} \end{array}$$ (5)

In Equations (3–5), t denotes the time dimension, covering the period from 1990 to 2021. The terms $\alpha_{0},\beta_{0}$ and $\lambda_{0}$ represent the constant terms of each equation. The coefficients $\alpha_{i},\beta_{i}$ and $\lambda_{i}$ (i = 1, 2, 3…5) indicate the elasticities of health outcomes with respect to the explanatory variables: gross domestic product (GDP), GDP squared (GDPSQ), greenhouse gas emissions (GHG), unemployment (UNEM), and economic globalization (KOF), respectively.

### 3.2 Data and descriptive statistics

This study examines the validity of the HKC hypothesis in Saudi Arabia over the period 1990 to 2021. The study employs a balanced dataset with no missing values or temporal inconsistencies, providing 32 consistent observations for all variables. The availability of consistent and reliable data primarily determines the timeframe. The dependent variable, public health, is measured using three indicators to capture different dimensions of health outcomes: overall disease burden (Disability-Adjusted Life Years, DALYs), mortality (Years of Life Lost, YLLs), and morbidity (Years Lived with Disability, YLDs). The empirical analysis incorporates five key explanatory variables: gross domestic product per capita (GDP), the square of GDP per capita (GDPSQ), environmental degradation, measured by greenhouse gas emissions (GHG), the unemployment rate (UNEM), and economic globalization (KOF). GDP and GDPSQ are included to examine the nonlinear connection between economic growth and public health, as stated by the HKC hypothesis. GHG serves as a proxy for environmental degradation, capturing the health risks associated with atmospheric pollution. The unemployment rate reflects socioeconomic stress, while the KOF index represents the extent of a country’s integration into the global economy. Data for GDP, GHG, and UNEM are sourced from the World Development Indicators of the World Bank. Public health indicators (DALYs, YLLs, and YLDs) are obtained from the Global Burden of Disease database compiled by the Institute for Health Metrics and Evaluation. Finally, the KOF economic globalization index is drawn from Dreher (51) and Gygli et al. (52). All variables were transformed into their natural logarithmic forms to mitigate potential heteroscedasticity and to facilitate the interpretation of estimated coefficients as elasticities. Table 1 presents detailed definitions and sources for all variables.

**Table 1:** Definitions and sources of variables.

Acronym	Variable	Definition	Source
ln DALYs	Overall disease burden	Disability-adjusted life years	GBD
ln YLLs	Mortality	Years of life lost due to premature mortality	
ln YLDs	Morbidity	Years lived with a disability	
ln GDP	Economic growth	GDP per capita (constant 2015 US$)	WDI
ln GDPSQ	Square of economic growth	GDP per capita squared	
ln GHG	Environmental degradation	Greenhouse gas emissions	
ln UNEM	Unemployment rate	The share of the labor force that is without work but available for and seeking employment (%).	
ln KOF	Economic globalization	KOF economic globalization index (trade and financial flows)	Dreher (51) and Gygli et al. (52)

Table 2 reports the descriptive statistics for the variables. All mean values are positive, indicating generally consistent trends across the dataset. The mean value of GHG emissions is 15.698, with a standard deviation of 0.396. In contrast, KOF shows a mean of 4.146 and a lower variability (SD = 0.054), reflecting relatively stable globalization trends. Similarly, the mean values for DALYs (15.698, SD = 0.193), YLLs (15.248, SD = 0.131), YLDs (14.667, SD = 0.332), GDP (10.023, SD = 0.073), GDPSQ (100.456, SD = 0.457), and UNEM (1.763, SD = 0.142) are all positive. We also examined the potential multicollinearity between explanatory variables through the computation of Variance Inflation Factors (VIF). As shown in Table 2, the maximum VIF value is 2.015, well below the conventional thresholds of 5. Therefore, multicollinearity among the explanatory variables does not pose substantial risks of inflating standard errors.

**Table 2:** Descriptive statistics and multicollinearity check.

Statistics	ln DALYs	ln YLLs	ln YLDs	ln GDP	ln GHG	ln UNEM	ln KOF
Mean	15.698	15.248	14.667	10.023	6.135	1.763	4.146
Median	15.627	15.217	14.627	10.017	6.150	1.750	4.138
Maximum	16.106	15.563	15.235	10.177	6.630	2.036	4.286
Minimum	15.515	15.101	14.149	9.887	5.466	1.469	4.052
Std. Dev.	0.193	0.131	0.332	0.073	0.396	0.142	0.054
VIF	–	–	–	2.015	1.176	1.658	1.195
Observations	32	32	32	32	32	32	32

**Figure 1:**
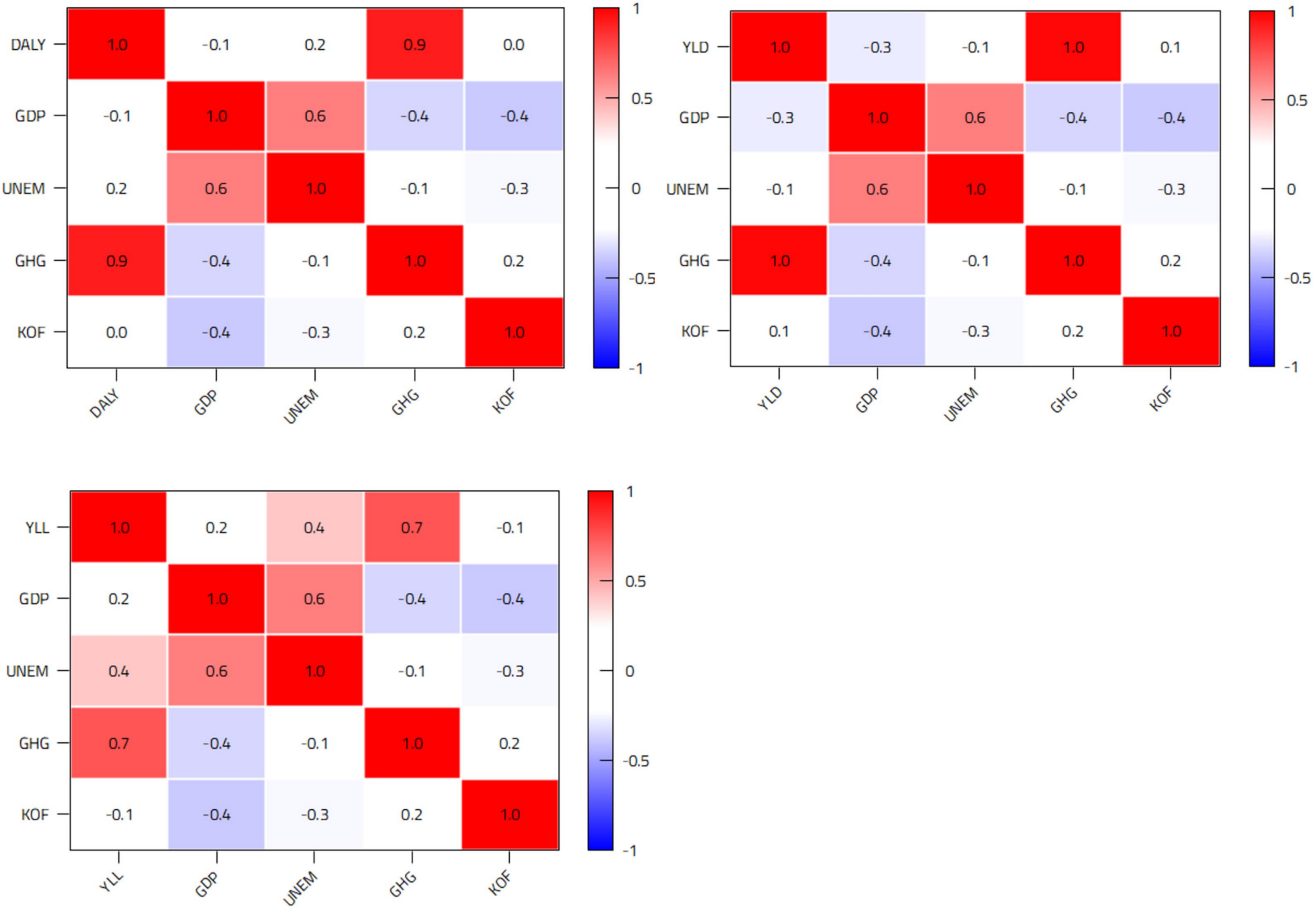
Correlation matrices.

Figure 1 reports the correlation analysis between the different variables. The results show the existence of negative associations between GDP and DALYs and their components, except YLLs. Moreover, the correlation between GDP and YLDs is generally greater than between GDP and DALYs.

### 3.3 Methodology

The ARDL model developed by Pesaran et al. (53) was proposed as a cointegration test with several advantages over conventional cointegration techniques. One key advantage of the ARDL model is that it can accommodate a mix of I(0) and I(1) variables. In addition, it estimates simultaneously the short-and long-run coefficients within a single reduced-form equation. Finally, the ARDL model yields more robust and reliable estimates in small samples, which is particularly relevant for the present study given the limited number of observations (32).

Despite these advantages, the linear ARDL model has some limitations. The main limitation is that it estimates the mean effects of explanatory variables on the dependent variable. The distributional characteristics of macroeconomic data, which are often non-normal and exhibit substantial heterogeneity, make the ARDL model less suitable for capturing dynamics across the entire distribution of the dependent variable. To address this limitation, the QARDL model, proposed by Cho et al. (21) provides short- and long-run coefficients at different quantiles of the distribution. In our case, the QARDL model assesses how economic growth and other explanatory variables affect public health across the distribution of health outcomes. We specifically employ the QARDL at three different quantiles: Q0.25, Q0.50, and Q0.75. The lower quantile (Q0.25) represents the lowest DALYs/YLLs/YLDs, indicating the best public health conditions, while the upper quantile (Q0.75) corresponds to the highest DALYs/YLLs/YLDs, reflecting the worst health outcomes. Finally, the median quantile (Q0.50) captures the effects of economic growth on public health at moderate health levels. Examining the effects of economic growth on public health outcomes at different levels of public health allows identifying whether the HKC hypothesis is confirmed and whether explanatory variables have significant effects during low, moderate, and high disease burden levels. This allows the policymakers to design and implement state-specific interventions to improve the public health outcomes.

The QARDL model, an extension of the standard ARDL model, allows for the examination of nonlinear relationships between the dependent and independent variables across different quantiles of the dependent variable. The ARDL model may be written in the following form, as shown in Equation (6):


$$\begin{array}{l} \mathrm{\Delta H}O_{t}^{m}=\alpha +\sum_{i=1}^{n1}\beta_{1i}\mathrm{\Delta H}O_{t-i}^{m}+\sum_{i=0}^{n2}\beta_{2i}\mathrm{\Delta GD}P_{t-i}\\ +\sum_{i=0}^{n3}\beta_{3i}\mathrm{\Delta GD}P_{t-i}^{2}+\sum_{i=0}^{n4}\beta_{4i}\mathrm{\Delta GH}G_{t-i}\\ +\sum_{i=0}^{n5}\beta_{5i}\text{ΔUNE}M_{t-i}+\sum_{i=0}^{n6}\beta_{6i}\mathrm{\Delta KO}F_{t-i}\\ +\beta_{7}HO_{t-1}^{m}+\beta_{8}\mathrm{GD}P_{t-1}+\beta_{9}\mathrm{GD}P_{t-1}^{2}+\beta_{10}\mathrm{GH}G_{t-1}\\ +\beta_{11}\mathrm{UNE}M_{t-1}+\beta_{12}\mathrm{KO}F_{t-1}+\omega_{t}^{m}\;\; \end{array}$$ (6)

where *Δ* is the first difference operator, α represents the constant term, and $\omega_{t}$ denotes the residual term. The coefficients of short-run relationship are denoted by $\beta_{1}$ , $\beta_{2}$ , $\beta_{3}$ , $\beta_{4}$ , $\beta_{5}$ and $\beta_{6}$ , while the coefficients of long-run relationship are denoted by $\beta_{7}$ , $\beta_{8}$ , $\beta_{9}$ , $\beta_{10}$ , $\beta_{11}$ , and $\beta_{12},$ respectively. The appropriate lag lengths for these differenced variables are selected based on the Akaike Information Criterion (AIC).

To examine the asymmetric effects of macroeconomic variables on health output, this study employs the QARDL model. This approach enables the analysis of long-run equilibrium relationships across different quantiles of the dependent variable. The QARDL model is specified as follows:


$$\begin{array}{l} Q_{\mathrm{\Delta H}O_{t}^{m}}=\alpha (\tau )+\sum_{i=1}^{n1}\beta_{1i}(\tau )\mathrm{\Delta H}O_{t-i}^{m}+\sum_{i=0}^{n2}\beta_{2i}(\tau )\mathrm{\Delta GD}P_{t-i}\\ +\sum_{i=0}^{n3}\beta_{3i}(\tau )\mathrm{\Delta GD}P_{t-i}^{2}+\sum_{i=0}^{n4}\beta_{4i}(\tau )\mathrm{\Delta GH}G_{t-i}\\ +\sum_{i=0}^{n5}\beta_{5i}(\tau )\text{ΔUNE}M_{t-i}+\sum_{i=0}^{n6}\beta_{6i}(\tau )\mathrm{\Delta KO}F_{t-i}\\ +\beta_{7}(\tau )HO_{t-1}^{m}+\beta_{8}(\tau )\mathrm{GD}P_{t-1}+\beta_{9}(\tau )\mathrm{GD}P_{t-1}^{2}\\ +\beta_{10}(\tau )\mathrm{GH}G_{t-1}+\beta_{11}(\tau )\mathrm{UNE}M_{t-1}+\beta_{12}(\tau )\mathrm{KO}F_{t-1}+\omega_{t}^{m}(\tau )\; \end{array}$$ (7)

where $\omega_{t}^{m}(\tau )=HO_{t}^{m}-Q_{HO_{t}^{m}}(\frac{\tau }{\omega_{t-1}^{m}})$ . Additionally, the range 0< τ <1 represents the quantiles. To incorporate the error correction mechanism (ECM) within the QARDL model, Equation (7) is reformulated into the generalized form presented in Equation (8) below:


$$\begin{array}{l} Q_{\mathrm{\Delta H}O_{t}^{m}}=\alpha (\tau )+\rho (\tau )HO_{t-1}^{m}-\beta_{8}(\tau )\mathrm{GD}P_{t-1}-\beta_{9}(\tau )\mathrm{GD}P_{t-1}^{2}\\ -\beta_{10}(\tau )\mathrm{GH}G_{t-1}-\beta_{11}(\tau )\mathrm{UNE}M_{t-1}-\beta_{12}(\tau )\mathrm{KO}F_{t-1}+\\ \sum_{i=1}^{n1}\beta_{1i}(\tau )\mathrm{\Delta H}O_{t-i}^{m}+\sum_{i=0}^{n2}\beta_{2i}(\tau )\mathrm{\Delta GD}P_{t-i}+\sum_{i=0}^{n3}\beta_{3i}(\tau )\mathrm{\Delta GD}P_{t-i}^{2}\\ +\sum_{i=0}^{n4}\beta_{4i}(\tau )\mathrm{\Delta GH}G_{t-i}+\sum_{i=0}^{n5}\beta_{5i}(\tau )\text{ΔUNE}M_{t-i}\\ +\sum_{i=0}^{n6}\beta_{6i}(\tau )\mathrm{\Delta KO}F_{t-i}+\varepsilon_{t}^{m}(\tau \;) \end{array}$$ (8)

where the error correction term ( ρ ) is expected to be negative and statistically significant, confirming the existence of a stable long-run equilibrium. As previously noted, it is essential to conduct a Wald test to assess the presence of significant differences across the quantiles for the various variables.

**Figure 2:**
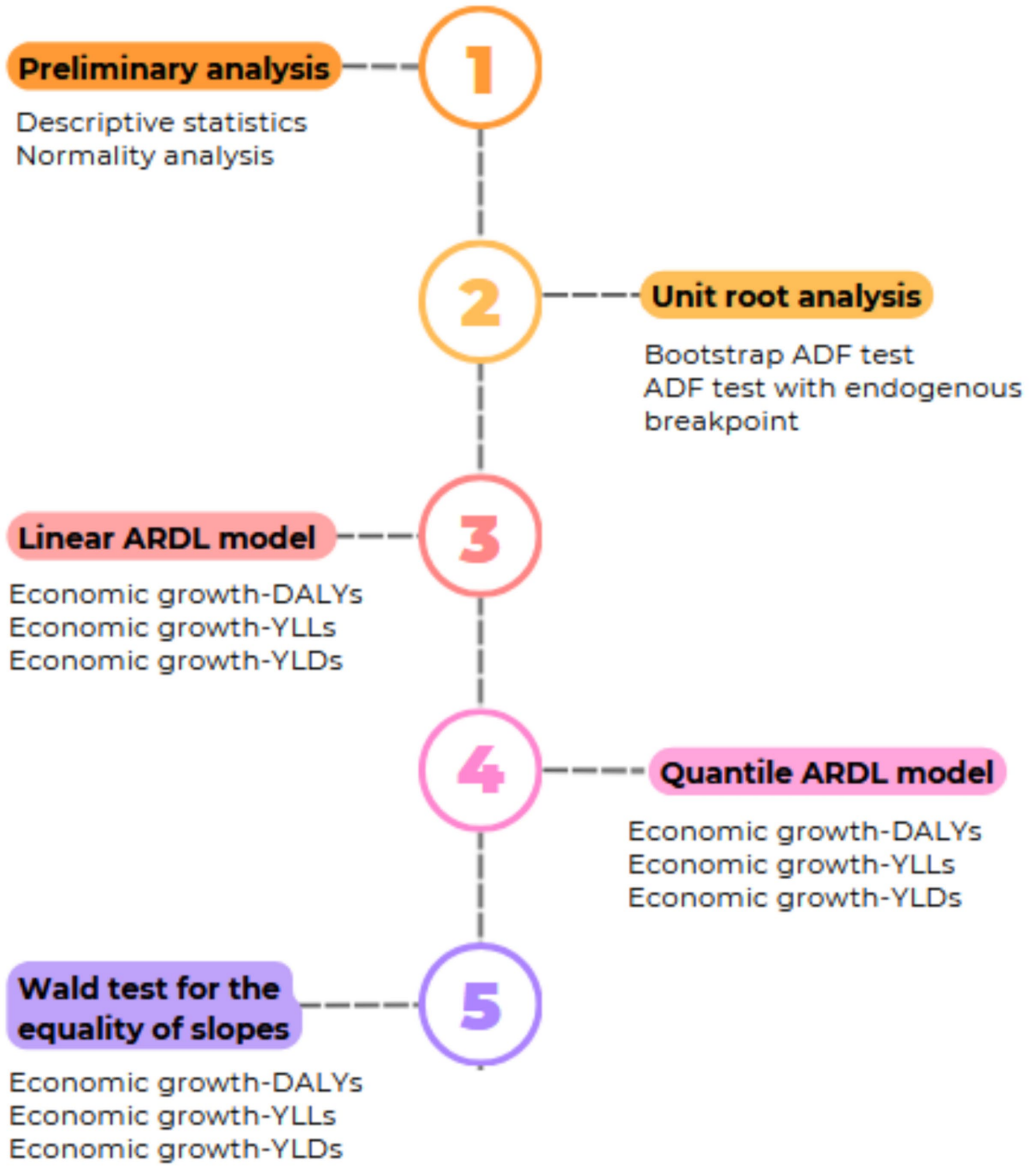
Empirical methodology.

As illustrated in Figure 2, the empirical analysis proceeds through multiple stages. We start by checking the distributional properties of dependent variables (DALYS, YLLs and YLDs) using both normality tests and graphical inspection. This is crucial for validating the suitability of the Quantile ARDL approach. Next, we conduct a stationarity analysis using both conventional unit root test and test that accounts for endogenous structural breaks. This step is essential to ensure that none of the variables are integrated of order two or higher. Then, we estimate the short- and long-run coefficients using the linear ARDL model across all three models. This serves as a benchmark for comparison. We next estimate the quantile ARDL model, and finally, the Wald test of equality of slopes is applied to assess whether the estimated parameters differ significantly across quantiles.

## 4 Results

### 4.1 Normality analysis

**Figure 3:**
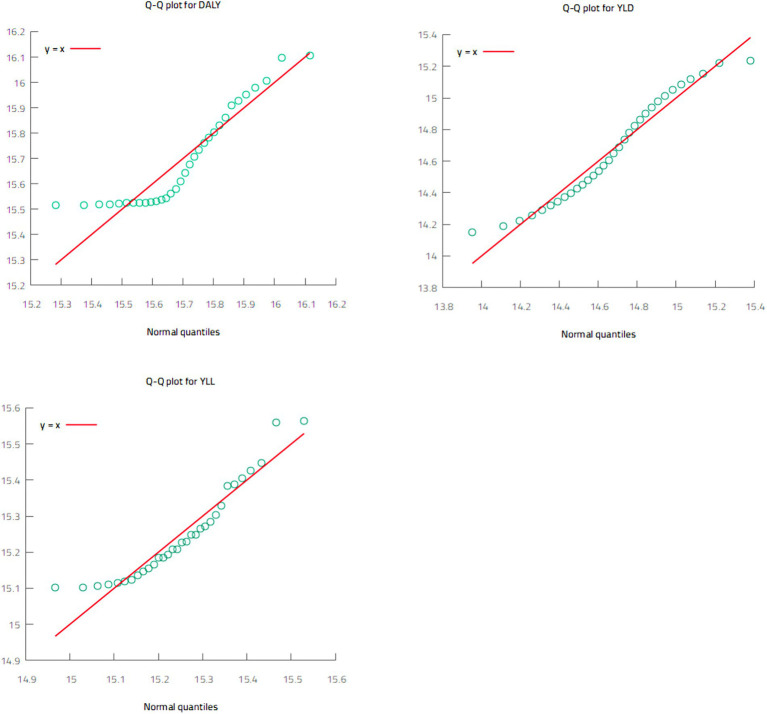
Q-Q plots of the dependent variables.

Before applying the QARDL model, it is important to examine the distributional properties of the dependent variable, as this model is particularly suited for non-normally distributed series. Indeed, when normality assumptions are violated, standard OLS-based methods, including the ARDL model, may yield biased results. To check normality, we employ the standard normality tests, specifically the Jarque-Bera, Shapiro–Wilk W and Shapiro-Francia W′ tests (Table 3), alongside a graphical examination of the distribution using the quantile-quantile (Q-Q) plots reported in Figure 3.

**Table 3:** Normality analysis.

Variables	Jarque-Bera test	Shapiro–Wilk W test	Shapiro-Francia W′ test
ln DALYs	4.630* (0.098)	0.847*** (0.000)	0.861*** (0.001)
ln YLLs	5.160* (0.075)	0.898*** (0.000)	0.906** (0.012)
ln YLDs	6.360** (0.041)	0.948*** (0.000)	0.963 (0.292)

***, **, and * indicate rejection of the null hypothesis of normality.

The results in Table 3 indicate that DALYs, YLLs, and YLDs are not normally distributed at different significance levels. Although the different tests provide different findings, there is consistent evidence that all three dependent variables deviate from normality. Regarding the Q-Q plots, a variable can be considered normally distributed if the green data points in Figure 3 closely align with the red diagonal line. As shown, the green data points significantly deviate from the diagonal in all cases, indicating that none of the dependent variables follow a normal distribution. Overall, the analysis confirms that the three dependent variables deviate from normality, supporting the suitability of the QARDL model for modelling the HKC hypothesis in Saudi Arabia.

### 4.2 Unit root analysis

Before implementing the ARDL and QARDL models, it is crucial to assess the stationarity of the data to ensure that no variables are integrated of order two or higher. The first test employed is the bootstrap ADF test of Park (54), an enhanced version of the standard ADF unit root test. Given its ability to provide more accurate critical values in small samples and to account for potential autocorrelation and heteroskedasticity, it offers improved reliability in detecting unit roots (55). Since the graphical analysis (not reported to save space) reveals some upward trends and potential structural breaks in the data, it seems important to apply unit root tests that accounts for structural changes. The ADF test with an endogenous breakpoint, using both the additive outlier (AO) and innovative outlier (IO) variants, is employed. While the additive outlier model captures sudden shifts in the level of a time series, reflecting immediate structural breaks, the innovative outlier model accounts for gradual changes in the series that evolve. Table 4 presents the findings of the different unit root tests for series at the level and first difference. The results suggest that all variables are integrated of order one, except GHG emissions, which have been found to be I(0) using the bootstrap ADF test. In addition, the three dependent variables are integrated of order one, an additional prerequisite for implementing the ARDL and QARDL models. The stationarity analysis confirms the suitability of ARDL/QARDL models for estimating the short- and long-run parameters.

**Table 4:** Unit root test results.

Variables	Bootstrap ADF		ADF with endogenous breakpoint			
	Statistics	Boot. crit. values	Innovative outliers		Additive outliers	
			Statistics	Break date	Statistics	Break date
Dependent variables						
DALYs	4.148	1.000	−1.572	2018	−3.543	2012
ΔDALYs	−3.470**	0.010	−5.019***	1999	−6.531***	2003
YLLs	2.576	1.000	−2.496	2014	−4.068	2009
ΔYLLs	−3.668**	0.010	−5.023***	1999	−6.078***	2003
YLDs	1.890	0.980	0.686	2005	−0.711	2018
ΔYLDs	−3.970**	0.020	−4.501**	1999	−6.453***	2005
Explanatory variables						
GDP	−1.941	0.400	−2.578	1993	−2.811	1992
ΔGDP	−5.221***	0.000	−6.526***	2002	−6.928***	2002
GDPSQ	−1.942	0.420	−2.587	1993	−2.811	1992
ΔGDPSQ	−5.218***	0.000	−6.529***	2002	−6.928***	2002
GHG	−2.153**	0.020	−1.395*	2002	−1.750	1997
ΔGHG	−4.001***	0.000	−6.375***	2017	−7.201***	2016
UNEM	−2.095	0.370	−3.364	2019	−3.327	2017
ΔUNEM	−5.461***	0.000	−6.820***	1999	−7.054***	1999
KOF	−1.732	0.570	−2.522	2013	−2.816	2008
ΔKOF	−4.538***	0.000	−4.902**	1994	−5.350***	2012

***, **, and * indicate rejection of the null hypothesis at the 1, 5, and 10% levels, respectively. For the ADF unit root test with endogenous breakpoint, critical values are −4.949(1%), −4.443(5%) and −4.193(10%). The Akaike Information Criterion (AIC) is used to identify the optimal lag length. All variables are expressed in their natural logarithmic form.

### 4.3 ARDL model estimation results

Table 5 displays the ARDL estimation results, highlighting the short- and long-run dynamics between the explanatory variables and public health indicators, namely DALYs, YLLs, and YLDs. In the long-run, the findings for DALYs and YLLs support the HKC hypothesis. Indeed, GDP and its squared term are statistically significant, with a positive coefficient on GDP and a negative coefficient on GDPSQ, suggesting an inverted U-shaped connection between economic growth and public health deterioration. This result aligns with those of Bai et al. (34), who reported that health outcomes initially worsen with economic expansion but begin to improve once a certain income threshold is surpassed. The turning points are estimated at $21059.453 for DALYs and $22165.672 for YLLs. In contrast, no turning point is identified for YLDs, given that both GDP and its square are statistically insignificant, indicating that economic growth does not influence health outcomes in the long-run. For DALYs and YLLs, the computed turning points reported below indicate the threshold level at which economic growth starts improving public health. However, before reaching the threshold levels, economic growth deteriorates both DALYs and YLLs. These outcomes strongly support the validity of the HKC hypothesis for the total health burden, as measured by DALYs, and the premature mortality, as measured by YLLs. However, no evidence of nonlinear effects of growth on morbidity, as measured by YLDs.

**Table 5:** Linear ARDL model results.

Variables	Dep. variable: lnDALYs		Dep. variable: lnYLLs		Dep. variable: lnYLDs	
	Coefficient	*p*-value	Coefficient	*p*-value	Coefficient	*p*-value
Panel A: Long-run effects						
ln GDP	122.370*	0.089	178.552*	0.066	871.304	0.332
ln GDPSQ	−6.146*	0.089	−8.921*	0.066	−43.713	0.332
ln GHG	0.681***	0.000	0.534***	0.000	0.982***	0.003
ln UNEM	0.701***	0.004	0.691***	0.001	3.964	0.334
ln KOF	0.122	0.641	−0.0003	0.999	6.495	0.317
Constant	−599.059*	0.095	−882.430*	0.070	−4363.929	0.333
Panel B: Short-run effects						
ECT	−0.126***	0.000	−0.161***	0.000	−0.013***	0.000
Δ ln GDP	28.789***	0.000	46.063***	0.000	16.446***	0.000
Δ ln GDP(−1)	/	/	/	/	−7.472***	0.000
Δ ln GDP(−2)	/	/	/	/	−0.024**	0.033
Δ ln GDPSQ	−1.451***	0.000	−2.321***	0.000	−0.827***	0.000
Δ ln GDPSQ(−1)	0.0009	0.432	/	/	0.376***	0.000
Δ ln GDPSQ(−2)	0.0003***	0.008	/	/	/	/
Δ ln GHG	0.112***	0.000	0.178***	0.009	−0.008	0.532
Δ ln GHG(−1)	−0.158***	0.000	−0.180***	0.001	−0.071***	0.000
Δ ln GHG(−2)	−0.251***	0.000	−0.228***	0.001	/	/
Δ ln UNEM	/	/	0.135***	0.000	0.048***	0.000
Δ ln UNEM(−1)	/	/	0.056***	0.000	−0.025***	0.000
Δ ln KOF	−0.028	0.212	−0.009	0.787	0.004	0.670
Δ ln KOF(−1)	0.078***	0.002	0.162***	0.000	/	/
Δ ln KOF(−2)	0.101***	0.000	0.190***	0.000	/	/
Panel C: Threshold level						
Turning point	9.9551		10.0063		NA	
Threshold level (US $)	21059.453		22165.672		NA	
Panel D: Validation tests						
F-statistics	81.301***		34.885***		35.717***	
J-B normality test	0.401		0.427		0.804	
LM test	0.258		0.295		0.061*	
ARCH test	0.183		0.442		0.792	
Adjusted R-squared	0.949		0.929		0.952	
CUSUM	S		S		S	

***, **, and * indicate the statistical significance at the 1, 5, and 10% levels, respectively. The Akaike Information Criterion (AIC) is used to identify the optimal lag length. “S” in Panel D stands for the stability of long-run coefficients using the CUSUM test. “NA” in Panel C indicates that no significant turning point was identified for this model, as GDP and/or its squared term were not statistically significant.

Greenhouse gas emissions and the unemployment rate show statistically significant positive effects on both DALYs and YLLs, indicating that environmental degradation and labor market distress contribute directly to worsening specific public health outcomes. These results are consistent with the findings of Mathieu et al. (56), Arena et al. (57), and Kiehbadroudinezhad et al. (58), who documented similar adverse effects of unemployment and pollution on public health. In contrast, globalization has no significant coefficient in the long-run, indicating no substantial role in influencing long-term health outcomes in Saudi Arabia. Our results are inconsistent with the results of Martens et al. (59) and Shobande et al. (60), which emphasized a significant association between globalization and mortality rates. Regarding YLDs, none of the variables exhibit long-run significance, suggesting that the drivers of morbidity may become more complex over time.

In the short-run (Panel B), the ARDL model provides negative and significant error correction terms (ECTs) across all three models. This confirms the presence of long-run cointegrating linkages, i.e., the system adjusts toward long-run equilibrium as a result of short-term shocks. For example, the ECT of −0.126 for DALYs indicates that any short-run disequilibrium is adjusted with a speed of around 12.6% toward the long-run equilibrium. This conclusion is also confirmed using the F-statistics reported at the bottom of Table 5 and indicating that the rejection of the null hypothesis of no cointegration at 1% level for all three models. GDP and GDPSQ maintain their significance in the short-run for all dependent variables, further reinforcing the nonlinear effect of economic growth. GHG emissions exhibit a detrimental and significant short-term impact on DALYs and YLLs, while unemployment shows a strong short-run association with both YLLs and YLDs. Interestingly, globalization displays some significant lagged impacts, especially on DALYs and YLLs, indicating that it only influences public health instantaneously but not in the long-run. The validity of the ARDL model has been checked based on a battery of diagnostic tests, with results reported at the bottom of Table 5. Overall, the tests suggest the absence of autocorrelation and heteroscedasticity in residuals, while confirming the normality and stability of the coefficients. Finally, all models exhibit high R-squared values, indicating a strong explanatory power of the independent variables.

Despite the ARDL model providing some fresh evidence on the validity of the HKC hypothesis in Saudi Arabia, it fails to account for nonnormal distribution of the dependent variables. To account for this issue and estimate the heterogeneous effects across the conditional distribution of health outcomes, the QARDL model is estimated.

### 4.4 Quantile ARDL model estimation results

This section provides estimations of the short- and long-run effects of the set of economic, social, and environmental factors across different quantiles (Q0.25, Q0.50, and Q0.75) of the public health using the QARDL model. Table 6 presents the results of the QARDL across the different quantiles of DALYs. The findings confirm a significant nonlinear relationship between economic growth and public health, thereby validating the HKC hypothesis across all quantiles. Specifically, GDP has a positive and significant effect on DALYs. At the same time, its squared term is negative and significant, indicating economic growth contributes to increased disease burden that at lower income levels. However, economic growth leads to health improvements once exceeding a given income threshold (ranging between $20,544 and $21,459). This inverted U-shaped relationship is consistent across all quantiles, suggesting the robustness of the HKC across various health levels. In other words, the HKC hypothesis holds whether DALYs is low, moderate or high. When checking the original dataset, one can assert that the threshold levels mentioned above have been exceeded in Saudi Arabia, which suggests that the country has entered the second stage of the HKC, wherein economic growth begins to reduce DALYs and enhance public health.

**Table 6:** Quantile ARDL model estimates for DALYs.

Variables	Q0.25		Q0.5		Q0.75	
	Coefficient	*p*-value	Coefficient	*p*-value	Coefficient	*p*-value
Panel A: Long-run effects						
ln GDP	137.758***	0.004	135.461*	0.090	105.096***	0.000
ln GDPSQ	−6.905***	0.004	−6.813*	0.089	−5.291***	0.000
ln GHG	0.523***	0.000	0.769***	0.000	0.795***	0.000
ln UNEM	0.653***	0.000	0.776***	0.007	0.647***	0.000
ln KOF	0.531***	0.000	−0.216	0.476	−0.402***	0.000
Constant	−677.601*	0.005	−662.449*	0.095	−510.163***	0.000
Panel B: Short-run effects						
ECT	−0.171***	0.000	−0.106***	0.000	−0.110***	0.000
Δ ln GDP	30.243***	0.000	29.650***	0.000	29.083***	0.000
Δ ln GDP(−1)	−0.644	0.926	/	/	0.041	0.263
Δ ln GDP(−2)	−13.542**	0.039	/	/	0.076*	0.077
Δ ln GDPSQ	−1.523***	0.000	−1.496***	0.000	−1.467***	0.000
Δ ln GDPSQ(−1)	0.030	0.931	0.001	0.229	/	/
Δ ln GDPSQ(−2)	0.0680**	0.038	0.004**	0.015	/	/
Δ ln GHG	−0.029	0.712	0.118**	0.020	0.071	0.262
Δ ln GHG(−1)	−0.271***	0.002	−0.142***	0.003	−0.157**	0.011
Δ ln GHG(−2)	−0.287***	0.001	−0.192***	0.000	−0.160**	0.015
Δ ln KOF	0.012	0.777	−0.046*	0.099	−0.053	0.150
Δ ln KOF(−1)	0.023	0.615	0.073**	0.017	0.103***	0.008
Δ ln KOF(−2)	0.072	0.147	0.136***	0.000	0.162***	0.000
Panel C: Threshold level						
Turning point	9.9739		9.9403		9.9303	
Threshold level (US $)	21459.450		20751.953		20543.805	
Panel D: Validation tests						
J-B	0.302		0.218		0.185	
LM test	0.211		0.177		0.303	
ARCH test	0.540		0.963		0.745	
Adjusted R-squared	0.653		0.768		0.675	
CUSUM	S		S		S	

***, **, and * indicate the statistical significance at the 1, 5, and 10% levels, respectively. The Akaike Information Criterion (AIC) is used to identify the optimal lag length. “S” in Panel D stands for the stability of long-run coefficients using the CUSUM test.

Moreover, greenhouse gas emissions have a positive coefficient across at all quantiles, revealing the adverse role of environmental degradation in deteriorating public health outcomes. Similarly, unemployment shows a significant detrimental long-run influence on DALYs across the distribution of the dependent variable, suggesting that labor market distress exacerbates the disease burden regardless of the initial health status. The effect of economic globalization (KOF), however, is found to be heterogeneous. At the lower quantile (Q0.25), globalization is positively and significantly associated with DALYs, possibly reflecting increased exposure to structural inequalities. At the median quantile (Q0.50), the effect is statistically insignificant, while at the upper quantile (Q0.75), globalization exhibits a negative and significant impact on DALYs, indicating that under higher disease burden, globalization may support improved health outcomes. In the short-run, the error-correction term is negative and significant across all quantiles, confirming the presence of a stable long-run cointegrating relationship between economic growth and DALYs. Short-run effects of GDP and GDPSQ are similar to their long-run patterns, thereby confirming the nonlinear association between the two variables and the validity of the HKC hypothesis. GHG emissions have adverse short-run effects at some quantiles, which may be due to the adaptation or mitigation efforts following environmental shocks. The short-run influence of globalization is more pronounced at higher quantiles, with significant lagged effects suggesting that its benefits may manifest over time.

Table 7 presents the QARDL model estimates across different quantiles of YLLs and YLDs. The long-run estimates (Panel A) reveal that economic growth has a significant and nonlinear impact on YLDs at all quantiles, while the nonlinear impact on YLLs is only confirmed for extreme quantiles (Q0.25 and Q0.75). Specifically, GDP is positively associated with YLLs and YLDs, while the squared GDP term is negative and significant, supporting the HKC hypothesis in most cases. This relationship suggests that at lower YLLs and YLDs levels, economic growth exacerbates health burdens. Nevertheless, growth contributes to reducing health-related losses after surpassing a turning point ranging between $21,450.163 and $21718.355 for YLLs and $19,810.870 and $20709.581 for YLDs. These findings reveal the limited range of the threshold levels for both YLLs and YLDs across the different quantile orders, thereby confirming the robustness of the quantile ARDL estimates. The effect of GHG is positive and significant across all quantiles in the long-run, affirming the detrimental impact of environmental degradation on both mortality and morbidity. Likewise, unemployment demonstrates a strong and statistically significant positive effect at all quantiles, reinforcing its role as a consistent driver of adverse health outcomes. Notably, globalization exhibits mixed long-run effects. For YLLs, it is positively significant at the lower quantile (Q0.25), insignificant at the median (Q0.5), and significantly negative at the upper quantile (Q0.75), implying that globalization might worsen mortality outcomes for the healthier population while improving them among more burdened groups. In contrast, globalization’s effect on YLDs is consistently positive and significant across all quantiles, suggesting that increased global integration may be associated with greater non-lethal health burdens due to occupational stress or shifting disease patterns.

**Table 7:** Quantile ARDL model estimates for Years of Life Lost (YLLs) and Years Lived with Disability (YLDs).

Variables	Dep. variable: lnYLLs						Dep. variable: lnYLDs					
	Q0.25		Q0.5		Q0.75		Q0.25		Q0.5		Q0.75	
	Coefficient	*p*-value	Coefficient	*p*-value	Coefficient	*p*-value	Coefficient	*p*-value	Coefficient	*p*-value	Coefficient	*p*-value
Panel A: Long-run effects												
ln GDP	157.365**	0.019	107.760	0.152	124.540***	0.000	382.413***	0.000	134.640*	0.069	295.636***	0.000
ln GDPSQ	−7.879**	0.019	−5.418	0.152	−6.243***	0.000	−19.246***	0.000	−6.804*	0.067	−14.873***	0.000
ln GHG	0.331***	0.000	0.555***	0.000	0.649***	0.000	0.848***	0.000	0.841***	0.000	0.818***	0.000
ln UNEM	0.740***	0.000	0.786***	0.008	0.718***	0.000	2.118***	0.000	1.012***	0.008	1.329***	0.000
ln KOF	0.537***	0.004	−0.371	0.342	−0.835***	0.000	3.397***	0.000	1.890***	0.004	3.404***	0.000
Constant	−775.832**	0.021	−523.522	0.162	−607.427***	0.000	−1906.191***	0.000	−665.144*	0.073	−1474.273***	0.000
Panel B: Short-run effects												
ECT	−0.207***	0.000	−0.143***	0.000	−0.144***	0.000	−0.025***	0.000	−0.047***	0.000	−0.030***	0.000
Δ ln GDP	40.529***	0.002	34.537***	0.000	36.641***	0.000	15.683***	0.000	15.293***	0.000	19.744***	0.000
Δ ln GDP(−1)	−0.032	0.976	/	/	/	/	−0.4886**	0.043	−2.996	0.123	−2.919	0.348
Δ ln GDP(−2)	−19.098*	0.069	/	/	/	/	/	/	/	/	/	/
Δ ln GDPSQ	−2.040***	0.002	−1.742***	0.000	−1.848***	0.000	−0.789***	0.000	−0.771***	0.000	−0.993***	0.000
Δ ln GDPSQ(−1)	0.011	0.983	0.002	0.313	−0.0005	0.825	0.246**	0.042	0.152	0.116	0.149	0.238
Δ ln GDPSQ(−2)	0.959*	0.068	0.007***	0.009	0.005*	0.071	/	/	/	/	/	/
Δ ln GHG	−0.062	0.628	0.151*	0.054	0.175*	0.058	0.007	0.773	−0.017	0.418	−0.031	0.249
Δ ln GHG(−1)	−0.321**	0.014	−0.199***	0.000	−0.070	0.362	−0.092***	0.000	−0.142***	0.000	−0.149***	0.000
Δ ln GHG(−2)	−0.375***	0.007	−0.314***	0.000	−0.255***	0.006	−0.061**	0.036	−0.078***	0.001	−0.041	0.129
Δ ln UNEM	/	/	/	/	/	/	0.047***	0.000	0.037***	0.000	0.030***	0.000
Δ ln UNEM(−1)	/	/	/	/	/	/	−0.034***	0.007	/	/	−0.018**	0.046
Δ ln KOF	0.021	0.766	−0.045	0.293	−0.083	0.109	−0.014	0.353	−0.009	0.444	0.003	0.857
Δ ln KOF(−1)	0.041	0.588	0.122***	0.000	0.206***	0.000	−0.036**	0.041	−0.021	0.133	/	/
Δ ln KOF(−2)	0.094	0.248	0.188***	0.000	0.222***	0.000	−0.0009	0.554	/	/	/	/
Panel C: Threshold level												
Turning point	9.9859		NA		9.9734		9.9347		9.8939		9.9383	
Threshold level (US $)	21718.355		NA		21450.163		20634.330		19810.870		20709.581	
Panel D: Validation tests												
J-B	0.115		0.271		0.183		0.221		0.305		0.107	
LM test	0.075*		0.117		0.200		0.389		0.237		0.535	
ARCH test	0.619		0.641		0.617		0.705		0.735		0.787	
Adjusted R-squared	0.767		0.710		0.791		0.765		0.836		0.702	
CUSUM	S		S		S		S		S		S	

***, **, and * indicate the statistical significance at the 1, 5, and 10% levels, respectively. The Akaike Information Criterion (AIC) is used to identify the optimal lag length. “S” in Panel D stands for the stability of long-run coefficients using the CUSUM test. “NA” in Panel C indicates that no significant turning point was identified for this model, as GDP and/or its squared term were not statistically significant.

The error correction terms reported in Table 7 are all negative and highly significant across all quantiles, which confirms a significant long-run cointegration relationship. GDP and its square maintain their significance in the short-run, indicating persistent nonlinear dynamics. GHG emissions display some negative short-run lagged effects, suggesting partial responses of health outcomes to environmental shocks. Unemployment shows a positive short-run effect on YLDs across all quantiles, with some lags having negative coefficients. Globalization exhibits positive and significant effects on YLLs at higher quantiles (Q0.5 and Q0.75), while its impact on YLDs is mostly insignificant. These variations highlight the time-sensitive and quantile-dependent nature of globalization on different health metrics. Panel C reports the threshold levels for the two public health metrics (YLLs and YLDs) across different quantile orders (Q0.25, Q0.5, and Q0.75). As can be seen, the threshold levels for YLLs ($21718.355 at Q0.25 and $21450.163 at Q0.75) are slightly higher than those associated with YLDs ($20634.330 at Q0.25, $19810.870 at Q0.5, and $20709.581 at Q0.75). Such findings suggest that economic growth starts to reduce non-fatal health burdens (disability) before reducing premature deaths in Saudi Arabia. This could be explained by the fact that relatively basic interventions, such as improved access to primary healthcare services, may effectively lower disabilities even at lower stages of economic development. However, reducing premature deaths, particularly from chronic diseases, often requires more developed health infrastructure, specialized medical services, and stronger public health systems.

Overall, the QARDL model results indicate that the relationships between the explanatory variables and public health outcomes, particularly YLLs and YLDs, are not only nonlinear but also vary across the conditional distribution of health outcomes. These findings support the need for quantile-sensitive and targeted public health interventions that address both the short- and long-run effects of macroeconomic and environmental factors. The diagnostic analysis reported at the bottom of Tables 6, 7 indicates no evidence of serial correlation, heteroskedasticity, or instability, thereby ensuring the reliability of the results across quantiles.

### 4.5 Wald test for equality of slopes

Following the estimation of the QARDL coefficients, one should assess whether the coefficients differ significantly across quantiles. To this end, we employ the Wald test for the equality of slope. Table 8 reports the results across different quantile pairs (Q0.25 vs. Q0.50; Q0.25 vs. Q0.75; and Q0.50 vs. Q0.75) for the three dependent variables. The results strongly reject the null hypothesis of slope equality across quantiles for most variables, particularly for DALYs and YLLs.

**Table 8:** Wald test for the equality of slopes.

Variables	Q0.25 against Q0.5		Q0.25 against Q0.75		Q0.5 against Q0.75	
	Test statistic	*p*-value	Test statistic	*p*-value	Test statistic	*p*-value
Dep. variable: lnDALYs						
ln GDP	14.783***	0.005	24.150***	0.000	28.913***	0.000
ln GDPSQ	14.703***	0.005	24.072***	0.000	28.802***	0.000
ln GHG	35.845***	0.000	53.308***	0.000	65.804***	0.000
ln UNEM	5.127**	0.023	14.736***	0.000	15.476***	0.000
ln KOF	34.008***	0.000	188.819***	0.000	82.716***	0.000
All variables	113.834***	0.000	94.891***	0.000	11.079	0.804
Dep. variable: lnYLLs						
ln GDP	15.004	0.000	29.032***	0.000	31.702***	0.000
ln GDPSQ	14.933***	0.004	29.010***	0.000	31.638***	0.000
ln GHG	28.693***	0.000	69.355***	0.000	73.415***	0.000
ln UNEM	5.656**	0.017	7.446***	0.006	8.960**	0.011
ln KOF	31.673***	0.000	71.399***	0.000	75.246***	0.000
All variables	91.145***	0.000	165.910***	0.000	188.033***	0.000
Dep. variable: lnYLDs						
ln GDP	1.342	0.719	9.121**	0.027	13.618**	0.034
ln GDPSQ	1.365	0.713	9.038	0.028	13.511**	0.035
ln GHG	3.064	0.547	16.061***	0.002	21.058***	0.007
ln UNEM	1.849	0.604	19.636***	0.000	22.438***	0.001
ln KOF	1.450	0.835	88.320***	0.000	102.645***	0.000
All variables	18.445	0.426	135.652***	0.000	180.224***	0.000

***, **, and * indicate rejection of the null hypothesis of slope equality across specific quantiles at the 1, 5, and 10% levels, respectively.

For DALYs, GDP and its squared term (GDPSQ), GHG, and UNEM show statistically significant slope differences across all quantile pairs, with *p*-values below 0.01 or 0.05. This indicates substantial heterogeneity in the influence of these variables on the health burden across the distribution. This confirms the suitability of the QARDL approach for the analysis. The coefficient of globalization (KOF) is also significantly different across quantiles, especially between Q0.25 and Q0.75 (χ^2^ = 188.819, p-value = 0.000), suggesting that globalization impacts health outcomes differently across quantiles. The joint Wald test for all variables is highly significant in the comparisons between Q0.25 and Q0.50, and Q0.25 and Q0.75. However, it becomes insignificant between Q0.50 and Q0.75 (p-value = 0.804), which imply that most of the differences occurs in the lower half of the health distribution. For YLLs, we observe the same pattern as DALYs. All macroeconomic, environmental and social variables show statistically significant slope differences across all quantile pairs. The joint Wald statistics for all variables are significant in every comparison. In contrast, the results for YLDs are more nuanced. While slope coefficients for GDP and GDPSQ are not significantly different between Q0.25 and Q0.50, they become significant in comparisons involving Q0.75, indicating that economic growth has a more pronounced impact on morbidity at higher levels of the health burden. Similarly, GHG and UNEM only show significant slope differences when comparing higher quantiles, suggesting that the adverse effects of environmental degradation and unemployment on morbidity are more noticeable during periods of high morbidity levels. Notably, KOF exhibits highly significant differences across all quantile comparisons, with the most substantial heterogeneity appearing between Q0.50 and Q0.75 (*χ*^2^ = 102.645, *p*-value = 0.000).

In summary, the Wald test results in Table 8 provide robust evidence that the effects of macroeconomic, environmental, and social variables on public health outcomes are quantile-dependent and vary across different levels of the health burden distribution. These findings justify the use of the QARDL model for analyzing the HKC hypothesis in Saudi Arabia.

## 5 Discussion

The findings of this study provide robust evidence supporting the Health Kuznets Curve hypothesis in Saudi Arabia. The results show that the relationship between economic growth and public health outcomes is nonlinear and asymmetric across different levels of disease burden. The positive and significant effect of GDP, followed by a negative and significant coefficient of its squared term, implies that at lower stages of economic development, growth exacerbates health burdens. At the same time, beyond a certain threshold, it contributes to health improvements. The QARDL model, confirms inverted U-shaped association is consistent across all quantiles and health indicators (DALYs, YLLs, and YLDs), highlighting the validity of the HKC hypothesis within the Saudi context. These findings are consistent with previous studies of Gangadharan et al. (61) and Costa-Font et al. (32), who confirm a health Kuznets’ curve on per capita income. The results also indicate that economic growth leads to a fall in DALYs and an improvement in health outcomes once exceeding a given income threshold (ranging between $20,544.805 and $21,459.450). The inverted U-shaped association is verified for all quantiles of DALYs, thereby indicating the robustness of the HKC across various health levels. In addition, the HKC hypothesis in confirmed for different levels of DALYs, low, moderate or high. It is worth noting that the threshold levels mentioned above have been already reached in Saudi Arabia, since GDP per capita exceeded $21,459.450. These findings suggest that the country has entered the second stage of the HKC, wherein economic growth begins to reduce DALYs and enhance public health.

The estimated turning points range from $19,810.870 to $21,718.355, highlighting minimal differences across health indicators and the consistency of the results. First, YLDs reaches the turning point ($19,810,870–$20709.581), followed YLLs ($21450.163–21718.355). The order of the turning points reflects the progressive nature of health improvements as income rises. The fact that YLDs reaches its turning point first suggests that early gains from economic growth are typically observed in reducing non-fatal health conditions. This stage often corresponds to improvements in basic healthcare infrastructure, preventive services, and living standards. As national income grows, people gain better access to sanitation, vaccination, nutrition, and primary healthcare, all of which help lower the prevalence and duration of disabling but non-lethal diseases. Second, YLLs achieves the turning point at early stages, which implies that reducing premature mortality requires more advanced and costly health interventions. Declines in YLLs often depend on specialized medical services, chronic disease management, road safety, and environmental protection, which emerge at higher levels of economic development. In other words, while economic growth first alleviates non-fatal conditions through improved living conditions and preventive care, it takes a more extended period of income growth to translate these benefits into longer life expectancy. This sequence (YLDs, then YLLs) illustrates a logic progress in the health transition process: societies first reduce disability, then achieve significant reductions in early mortality rates. Similar dynamics have been noted by Santana and dos Santos (62), who reported that improvements in health precede declines in mortality in Brazil.

The results also reveal that environmental degradation exerts a detrimental impact on all health burden indicators across both short- and long-term. This result aligns with Majeed and Ozturk (36), who found that environmental degradation deteriorates population health. This suggests that environmental quality remains a crucial determinant of population health, as pollution-induced diseases impose considerable morbidity and mortality costs. The quantile analysis further highlights that this adverse effect is persistent across all health levels, underscoring the need for stronger environmental regulations and investments in green technologies to mitigate health risks. These results confirm the findings of Kelly and Fussell (63) and Li et al. (68), which highlighted that emission-related diseases impose serious health costs. Likewise, the results indicate that unemployment has a positive impact on all health indicators across quantiles. This confirms that joblessness contributes to deteriorating physical and mental health due to economic insecurity, stress, and reduced access to healthcare. These findings align with Byaro et al. (64), who argued that unemployment is linked to poor health and higher mortality rates in Sub-Saharan Africa. However, our results are not in line with Ruhm (65, 66), who found that recessions and high unemployment do not harm population health. The effect of economic globalization is more heterogeneous. At lower quantiles, globalization significantly increases disease burden, suggesting that initial exposure to global markets may amplify health inequalities and environmental stress. However, at higher quantiles, globalization exerts a negative and significant impact on DALYs and YLLs, indicating that greater integration into the world economy improves health outcomes by facilitating access to advanced medical technologies and healthcare innovations. These mixed results are consistent with the dual perspective presented by Martens et al. (59), who found that the impact of globalization on mortality rates is mixed. In contrast, globalization is positively correlated with YLDs at all quantiles. These results suggest that greater integration into the global economy may increase the burden of non-fatal health conditions. This outcome is in line with the study of Barbalat et al. (67), who found that countries with the highest level of globalization experienced 31% increase in the burden compared to those with low level of globalization.

## 6 Conclusion

### 6.1 Summary of the findings

This study examined the validity of the HKC hypothesis in Saudi Arabia by analyzing the nonlinear relationship between GDP and public health outcomes, measured through Disability-Adjusted Life Years, Years of Life Lost, and Years Lived with Disability, and accounting for additional environmental and social factors. The study employs annual data from 1990 to 2021 and applies the linear ARDL and Quantile ARDL models.

The ARDL model suggests the validity of the HKC hypothesis for both DALYs and YLLs, but not for YLDs, with threshold levels of $21059.453 and $22165.672, respectively. Therefore, a nonlinear inverted U-shaped relationship between economic growth and specific health outcomes metrics (DALYs and YLLs) has been confirmed. Given the limitations of the linear ARDL, which does not account for the distribution of the dependent variable and provides o0nly the mean effects, we move to estimate the Quantile ARDL model. This analysis also indicates an inverted U-shaped relationship between income and all public health outcomes, supporting the existence of the HKC in the Saudi context. The threshold level from which income begins to reduce DALYs is found to range between $20543.805 and $21459.450. The QARDL findings also reveal an inverted U-shaped relationship for YLLs and YLDs. However, the threshold levels for YLLs are higher than those associated with YLDs, suggesting that economic growth starts to reduce non-fatal health burdens before reducing premature deaths in Saudi Arabia. Therefore, the QARDL model allowed to uncover heterogeneity across the distribution of health outcomes, revealing that the impacts of macroeconomic, environmental and social variables vary significantly across different levels of the public health burden. GHG emissions and unemployment lead to a deterioration in health across all quantiles, while the impact of globalization is found to be mixed. The Wald test for equality of slopes provides robust evidence that the effects of macroeconomic, environmental and social variables on public health outcomes are quantile-dependent and asymmetric. In addition, the diagnostic analysis confirms the validity of the findings obtained from the ARDL and QARDL models.

### 6.2 Policy implications

The results of this study yield important policy implications for health and economic planning programs within Saudi Arabia. First, the findings indicate that the estimated threshold levels have already been exceeded, suggesting that Saudi Arabia is currently positioned in the second stage of the HKC, where economic growth is associated with improvements in public health outcomes. These findings suggests that continued investment in sustainable economic development can yield further health gains, particularly if growth is accompanied by public health spending and improvements in healthcare services. In addition, policymakers should prioritize inclusive growth strategies that reinforce these health benefits, ensuring that rising national income translates into equitable health improvements across all population segments. Second, the threshold level for YLDs is found to be lower than that for YLLs, indicating that economic growth begins to reduce non-fatal health burdens before reducing premature mortality in Saudi Arabia. This implies that while income improves both the burden of disease-related disability (YLDs) and the burden from premature death (YLLs), the most pronounced impact is observed for non-fatal health burdens, as indicated by the higher coefficients and lower threshold level associated with YLDs. In this case, policymakers should focus on strengthening the capacity of the healthcare system to manage chronic conditions, investing in early detection, and enhancing the accessibility of emergency and specialized care. At the same time, addressing non-fatal conditions, such as mental health services and rehabilitation, is essential to promote public health. Third, the detrimental role of environmental degradation on public health underscores the need for integrating environmental sustainability into economic policy reforms. Controlling emissions and shifting the energy mix toward cleaner energy sources may yield significant public health benefits. At the same time, implementing stringent environmental policies, enforcing regulatory frameworks, and monitoring industrial activity are also essential to mitigating the adverse health impacts of environmental degradation. Finally, the positive association between unemployment and health burdens requires the implementation of active labor market policies to reduce unemployment, particularly among vulnerable populations.

### 6.3 Limitations and avenues for future research

Despite the study provides some novel insights into the growth-health nexus in Saudi Arabia, it has some limitations. First, the use of annual data over a relatively short period of 32 years may represent a limitation, despite the ARDL and Quantile ARDL are suited for small sample size. Higher-frequency data could provide better insights into the nonlinear relationships between economic growth and public health outcomes. Second, despite the QARDL model captures nonlinearity and asymmetry across quantiles, it does not address structural breaks occurred during the study period, which may influence results. Future studies may rely on regime-switching models that explicitly account for changes in the underlying data-generating process. In addition, estimating the effects of positive and negative shocks in economic growth on public health outcomes could provide interesting results. This analysis could be conducted using the nonlinear ARDL model. Third, the use of aggregate public health measures may hide important demographic differences in how economic and environmental factors may affect public health for specific groups of the population, such as females and youth. Finally, the present study focuses only on Saudi Arabia, which limits the generalizability of the results to other countries. Future studies could apply extend the analysis to other countries or regions.

## Data Availability

Publicly available datasets were analyzed in this study. This data can be found at: https://databank.worldbank.org/source/world-development-indicators.
